# Phytoestrogen Bakuchiol Exhibits *In Vitro* and *In Vivo* Anti-breast Cancer Effects by Inducing S Phase Arrest and Apoptosis

**DOI:** 10.3389/fphar.2016.00128

**Published:** 2016-05-24

**Authors:** Li Li, Xueping Chen, Chi C. Liu, Lai S. Lee, Cornelia Man, Shuk H. Cheng

**Affiliations:** ^1^Department of Biomedical Sciences, City University of Hong KongHong Kong, China; ^2^Vitargent (International) Biotechnology LimitedHong Kong, China; ^3^Department of Applied Biology and Chemical Technology, Hong Kong Polytechnic UniversityHong Kong, China

**Keywords:** bakuchiol, estrogenic activity, S phase arrest, apoptosis, breast cancer cell, medaka, zebrafish

## Abstract

Phytoestrogen has been proposed as an alternative to hormone replacement therapy, which has been demonstrated to promote a high risk of breast cancer. However, the effect of phytoestrogen on breast cancer development has not been fully understood. Bakuchiol is an active ingredient of a traditional Chinese herbal medicine *Fructus Psoraleae*, the dried ripe fruit of *Psoralea corylifolia* L. (Fabaceae). The *in vitro* and *in vivo* estrogenic activities and anti-breast cancer effects of bakuchiol have not been well-studied. We found that bakuchiol induced the GFP expression in transgenic medaka (*Oryzias melastigma*, Tg, Chg:GFP) dose-dependently (0–1 μg/ml), demonstrating its *in vivo* estrogenic activity. Low dose of bakuchiol (1 μg/ml) induced the cell proliferation and ERα expression in MCF-7 cells, which could be blocked by the anti-estrogen ICI 182780, suggesting the *in vitro* estrogenic activity of bakuchiol. Our data indicated that high doses of bakuchiol (>2 μg/ml) inhibited breast cancer cell growth, with a stronger anti-proliferative effect than resveratrol, a widely studied analog of bakuchiol. High doses of bakuchiol (4, 7, and 10 μg/ml) were used for the further *in vitro* anti-breast cancer studies. Bakuchiol induced ERβ expression and suppressed ERα expression in MCF-7 cells. It also induced S phase arrest in both MCF-7 and MDA-MB-231 cells, which could be rescued by caffeine. Knock-down of p21 also marginally rescued S phase arrest in MCF-7 cells. The S phase arrest was accompanied by the upregulation of ATM, P-Cdc2 (Tyr15), Myt1, P-Wee1 (Ser642), p21 and Cyclin B1, suggesting that blocking of Cdc2 activation may play an important role in bakuchiol-induced S phase arrest. Furthermore, bakuchiol induced cell apoptosis and disturbed mitochondrial membrane potential in MCF-7 cells. The bakuchiol-induced apoptosis was associated with increased expression of Caspase family and Bcl-2 family proteins, suggesting that bakuchiol may induce apoptosis via intrinsic apoptotic pathway. The *in vivo* anti-breast cancer effect of bakuchiol was further proved in zebrafish (*Danio rerio*, wild-type AB) xenografts. 0.5 μg/ml of bakuchiol significantly reduced the MCF-7 cell mass in zebrafish xenografts. Overall, these results suggested the potential of using bakuchiol in HRT and breast cancer treatment.

## Introduction

According to the statistics released by the International Agency for Research on Cancer (2013)^1^, 1.7 million women were diagnosed with breast cancer, which killed more than 0.5 million women in 2012. The incidence rate and mortality had been increased by 20 and 14% respectively since 2008 to 2012. Breast cancer incidence in developed countries of North America and Western Europe is historically higher than in Asian countries, which has been associated to the higher intake of phytoestrogens in the eastern continent (de Kleijn et al., 2001).

Phytoestrogens are a group of plant-derived substances, with an estradiol-like structure or function. The main phytoestrogen groups are lignans, flavonoids, coumestans, and stilbenes. Interest in phytoestrogen has also increased as a result of the concerns about HRT. HRT is widely used to relieve the menopausal symptoms of post-menopausal women. However, it has been demonstrated that estrogen, which is used in HRT, increases the risk of breast cancer development (Key et al., 2001). Thus, women, mainly those with a history of breast cancer, are turning to the use of phytoestrogens, in the belief that they protect them from breast cancer and they are safer than estrogen itself.

Selective estrogen receptor modulators are compounds that selectively interact with ER either as ER agonists or antagonists in different organs. They have been studied in the past decade to treat hormone responsive cancer and menopausal symptoms. Phytoestrogens have been proposed as natural SERMs (Oseni et al., 2008). Lignans are the most major phytoestrogens found in many fiber-rich foods such as cereal brans, fruits and vegetables. They possess weak estrogenic or anti-estrogenic activities (Pianjing et al., 2011). Soy isoflavones, such as genistein and daidzein, are the most widely studied phytoestrogens. These isoflavones have varying estrogenic and anti-carcinogenic activities, and they have been proposed as natural SERMs (Setchell, 2001). There are also many studies on coumestans. Coumestans are found in various beans, alfalfa, and clover sprouts. Many of their biological effects can be attributed to their activation of ER signaling pathway, and the anti-cancer effects of these compounds have been also described in the last decade (Nehybová et al., 2014). Resveratrol, found naturally in grapes and red wine, is the most common stilbene. It has gained considerable attention because of its chemopreventive, anti-oxidative, and anti-inflammatory properties (Patisaul and Jefferson, 2010). Although, there are many *in vitro* and *in vivo* data on the role of phytoestrogens in breast cancer treatment, the data needs further research.

Bakuchiol is a meroterpene, which can be found in the traditional Chinese herbal medicine *Fructus Psoraleae*, the dried ripe fruit of *Psoralea corylifolia* L. (Fabaceae). It is shown to have anti-microbial, anti-inflammatory, anti-oxidative, anti-osteoporosis, and anti-depression or anti-stress activities (Lim et al., 2009; Choi et al., 2010; Kim et al., 2013; Huang et al., 2014; Mao et al., 2014). The estrogenic activities of bakuchiol were reported in several *in vitro* models (Xin et al., 2010; Lim et al., 2011; Mao et al., 2014). However, whether bakuchiol displays estrogenic activity in *in vivo* model is rarely studied (Shou et al., 2007; Lim et al., 2009). We developed transgenic medaka (*Oryzias melastigma*, Tg, Chg:GFP), in which the liver-specific and estrogen-sensitive choriogenin gene promoter drives GFP expression, in a previous study (Chen et al., 2007). With this transgenic fish, we could screen estrogenic activity of bakuchiol *in vivo*. The anti-cancer potential of bakuchiol has been reported in very few studies. Bakuchiol inhibits liver cancer cell growth through inducing S phase arrest, caspase 9/3 activaton, p53 and Bax up-regulation, as well as Bcl-2 down-regulation (Chen Z. et al., 2010). It also inhibits human carboxylesterase 2, which is commonly expressed in tumor tissue and involved in the metabolism of endogenous lipids and drugs (Li et al., 2015). To date, limited studies have demonstrated the effect of bakuchiol on breast cancer cell growth (Chen H.L. et al., 2010; Xin et al., 2010). However, the anti-cancer effect of resveratrol, an analog of bakuchiol, has been extensively reported (Bernhard et al., 2000; Joe et al., 2002; Tyagi et al., 2005; Chen et al., 2013). The mechanisms of the anti-cancer effects of resveratrol, including promoting cell cycle arrest, inducing apoptosis, preventing tumor-derived nitric oxide synthase expression, inhibiting cyclooxygenase activity, and decreasing DNA binding activity of nuclear factor κB, have been well-summarized in a previous review (Carter et al., 2014). However, the mechanisms of how bakuchiol affects breast cancer cell growth and the *in vivo* anti-breast cancer effects of bakuchiol have not been investigated. It is important to investigate the estrogenic and anti-breast cancer activities of bakuchiol both *in vitro* and *in vivo*, and to reveal the underlying mechanisms of these effects.

## Materials and Methods

### Chemicals and Reagents

**FIGURE 1:**
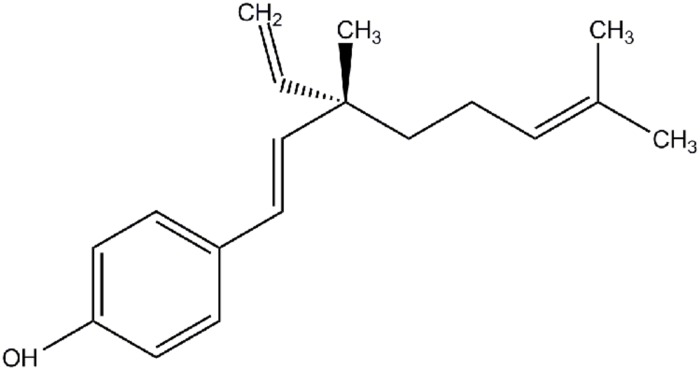
**Structure of bakuchiol**.

Bakuchiol {4-[(1*E*,3*S*)-3-ethenyl-3,7-dimethylocta-1,6-dienyl] phenol} (**Figure 1**) purity 98% by HPLC, was obtained from Enzo (Farmingdale, NY, USA). Resveratrol, 17β-estradiol (E2), and PTU was purchased from Sigma (St. Louis, MO, USA). ICI 182780 was bought from Abcam (Cambridge, MA, USA). DMEM, FBS, penicillin, streptomycin, PI, JC-1 dye, TRIzol reagent and PBS were purchased from Invitrogen (Carlsbad, CA, USA). FuGENE 6 transfection reagent, MTT, DMSO, caffeine, RNase A, annexin V-FITC/PI apoptosis kit, RQ1 RNase-free DNase, RIPA buffer, PMSF and Na_2_CO_3_ were bought from Promega (Madison, WI, USA). Kapa SYBR FAST qPCR kit was obtained from Kapa Biosystems (Wilmington, MA, USA), and high capacity RNA-to-cDNA master mix came from Applied Biosystems (Carlsbad, CA, USA). ECL Prime Western blotting detection system was obtained from GE Healthcare (Milwaukee, WI, USA). All antibodies were purchased from Cell Signaling Technology (Danvers, MA, USA).

### Medaka Fish Exposure

Larvae within 2 days dph were used for exposure. Twenty larvae were exposed in a 50 mm glass Petri dish for 24 h. Triplicates were carried out for each concentration and for the solvent control. After exposure, the fish were deposited onto the inner side of Petri dish cover with little water left. The induced GFP expression in the liver was observed under stereomicroscope (model M205 FA, Leica Microsystems, Germany). The images were recorded with the same parameter settings for the same set of exposed samples. Fluorescence intensity was quantified by image analysis software, Metamorph 5.0 (Universal Imaging, Downingtown, PA, USA). To reduce the interference of fish auto-fluorescence, only the fish liver was selected for analysis. The average fluorescence of the liver area was regarded as the GFP signal intensity. All procedures with fish were conducted following the License to Conduct Experiments approved by the Government of the Hong Kong SAR Department of Health [Ref. (21) in DH/HA&P/8/1/1 Pt.3].

### Cell Culture

Two breast cancer cell lines were used in this study. MCF-7 (ATCC: HTB-22) and MDA-MB-231 (ATCC: HTB-26) were purchased from American Type Culture Collection (ATCC, Manassas, VA, USA), The cell lines were routinely maintained in DMEM supplemented with 10% FBS, 0.37% Na_2_CO_3_, 50 units/ml penicillin and 50 μg/ml streptomycin, incubated at 37°C in a humidified atmosphere with 5% CO_2_.

### RNA Interference

Cells were seeded in 6-well plate and T25 flask, and allowed to attach before transfection. Cells were transfected with 25 nM siRNA (Ambion, Austin, TX, USA) with FuGENE 6 transfection reagent following the manufacturer’s recommendations. After transfection for 24 h, medium was changed and cells were incubated for 24 h before collected for following experiment.

### Cell Growth Inhibition Study Using MTT Assay

Cells were seeded at 5000 cells/well into 96-well plates. For the experiment in **Figure 3A**, MCF-7 and MDA-MB-231 cells were treated with serial concentrations of bakuchiol for 24, 48, and 72 h. For the experiment in **Figure 3B**, MCF-7 and MDA-MB-231 cells were treated with serial concentrations of resveratrol for 24, 48, and 72 h. For the experiment in **Figure 3C**, MCF-7 cells were treated with ethanol alone or 1 μg/ml bakuchiol and/or 1 μM ICI 182780 for 72 h. For the experiment in **Figure 6A**, MCF-7 cells were transfected with siCtrl or sip21, and treated with 0 or 7 μg/ml of bakuchiol for 24 h. After treatment, the cells were washed with PBS and incubated with 0.5% MTT for 3 h. DMSO was used to dissolve the formazan crystals, and the optical density of 570 nm of each well was obtained by a multi-well scanning spectrophotometer (Model 550, Bio-Rad, Hercules, CA, USA).

### Cell Cycle Analysis Using PI Staining

For the experiment in **Figure 4A**, MCF-7 cells and MDA-MB-231 cells were treated with varying concentrations of bakuchiol for 24 h. For the experiment in **Figure 5A**, MCF-7 cells and MDA-MB-231 cells were treated with ethanol alone or 7 μg/ml of bakuchiol and/or 5 mM of caffeine for 24 h. For the experiment in **Figure 6B**, MCF-7 cells were transfected with siCtrl or sip21, and treated with 0 or 7 μg/ml of bakuchiol for 24 h. After treatment, cells were trypsinized, washed with ice-cold PBS, and fixed with 70% ethanol at 4°C overnight. For staining, the fixed cells were centrifuged to remove ethanol and washed with PBS. Then the cell pellets were resuspended with 50 μg/ml PI and 100 μg/ml RNase A. After 30 min of incubation at 37°C, the stained cell suspension was analyzed by using a flow cytometer (Becton Dickinson, San Diego, CA, USA) with an excitation wavelength of 488 nm. The cell cycle distribution was calculated by using MODFIT 3.0 software (Verity Software House, Topsham, ME, USA).

### Examination of Bakuchiol-Induced Apoptosis with Annexin V/PI Staining

For the experiment in **Figure 7B**, MCF-7 cells were treated with 0–10 μg/ml of bakuchiol for 24 h. The analysis of apoptosis was conducted using annexin V-FITC/PI apoptosis kit in accordance with the manufacturer’s protocol.

### Measurement of Mitochondrial Membrane Potential with JC-1 Staining

For the experiment in **Figure 7C**, MCF-7 cells were treated with 0–10 μg/ml of bakuchiol for 24 h. Changes in mitochondrial membrane potential were measured by using a flow cytometer with JC-1 dye in accordance with the instructions of the manufacturer.

### RNA Extraction, Reverse Transcription, and Quantitative Real-Time PCR

**FIGURE 2:**
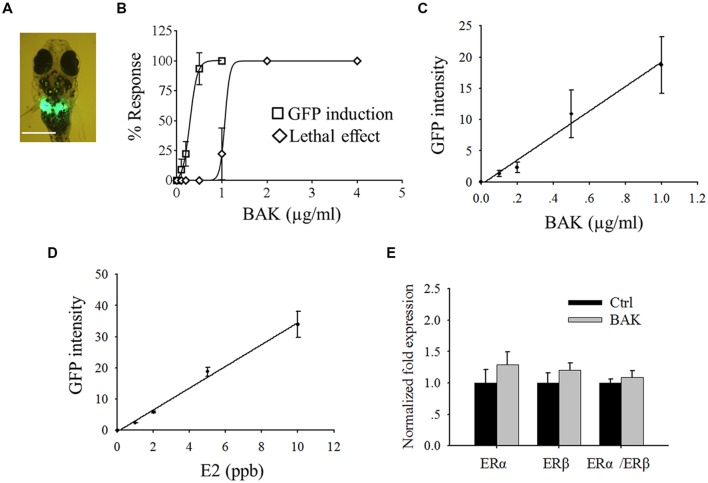
**Screening of *in vivo* estrogenic activity with transgenic medaka. (A)** Bakuchiol induced GFP expression in liver, the white bar indicates 100 μm. **(B)** LC_50_ and EC_50_ of fish after exposure to bakuchiol for 24 h. Correlation between different concentrations of **(C)** bakuchiol and **(D)** E2, and the induced GFP signal intensity. **(E)** Analysis of mRNA expression of ERα and ERβ by using real-time PCR. Fish (*n* = 10) were exposed to 0.5 μg/ml bakuchiol for 24 h. 18S rRNA was used as the internal reference. BAK, bakuchiol.

**FIGURE 3:**
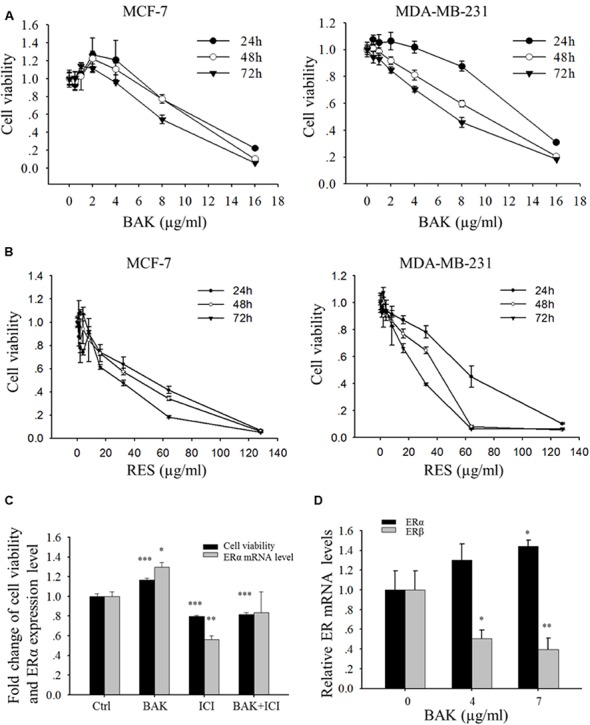
**Effects of bakuchiol on cell viability of breast cancer cells.** MCF-7 and MDA-MB-231 cells were treated with serial concentrations of **(A)** bakuchiol and **(B)** resveratrol for 24, 48 and 72 h, and cell proliferation was monitored by using MTT assay. The data were expressed as fold change of viability compared to the vehicle control (ethanol) with standard deviation. **(C)** Fold changes of cell viability and ERα mRNA levels of MCF-7 cells, which were treated with ethanol alone or 1 μg/ml bakuchiol and/or 1 μM ICI 182780 for 72 h (compared with control group, ^∗^*p* < 0.05, ^∗∗^*p* < 0.01, ^∗∗∗^*p* < 0.001, one-way ANOVA). **(D)** ERα and ERβ mRNA levels of MCF-7 cells, which were treated with 0, 4, or 7 μg/ml of bakuchiol for 24 h (compared with control group,^∗^*p* < 0.05, ^∗∗^*p* < 0.01, ^∗∗∗^*p* < 0.001, one-way ANOVA). BAK, bakuchol; RES, resveratrol; ICI, ICI 182780; Ctrl, control.

**FIGURE 4:**
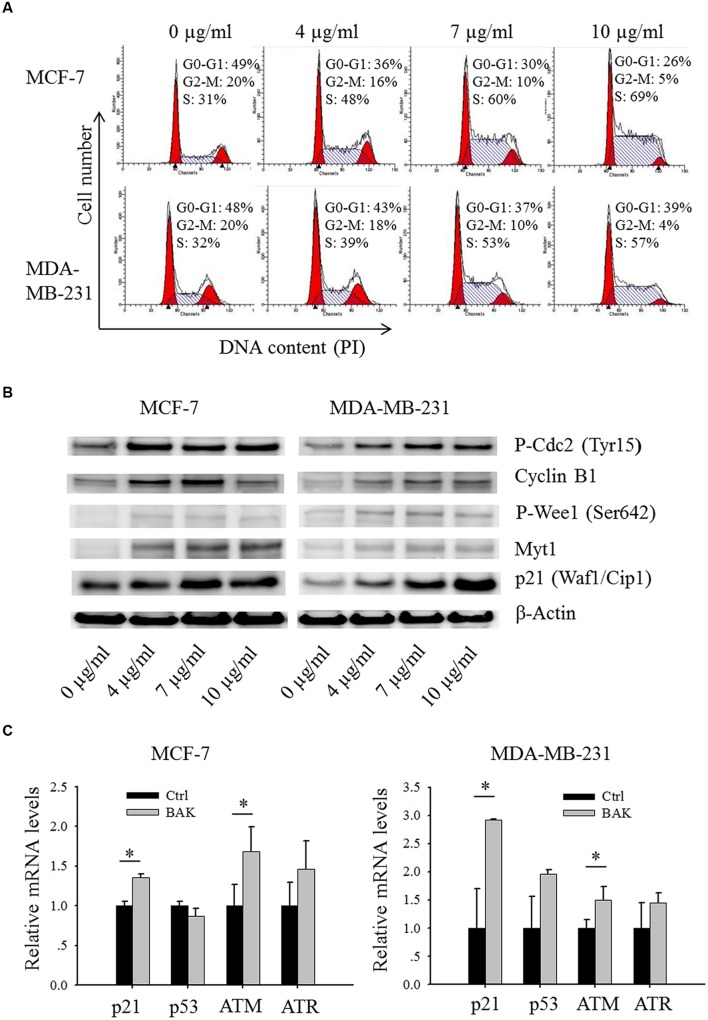
**Bakuchiol induced alteration in cell cycle and cell cycle regulator expression in breast cancer cells. (A,B)** Cells were treated with varying concentrations of bakuchiol as labeled in the figure for 24 h. **(A)** Cell cycle analysis with PI staining. **(B)** Expression levels of P-Cdc2 (Tyr15), Cyclin B1, P-Wee1 (Ser 642), Myt1, p21 (Waf1/Cip1) and β-Actin analyzed with Western blot **(C)** p21, p53, ATM and ATR mRNA expressions in cells treated with ethanol or 7 μg/ml of bakuchiol for 24 h. The data represent the mean ± SD of the values from three separate RNA samples (^∗^*p* < 0.05, student’s *t*-test). Ctrl, control; BAK, bakuchol.

For the experiment in **Figure 2E**, medaka fish (*n* = 10) were exposed to 0.5 μg/ml bakuchiol for 24 h. For the experiment in **Figure 3C**, MCF-7 cells were treated with ethanol alone or 1 μg/ml bakuchiol and/or 1 μM ICI 182780 for 72 h. For the experiment in **Figure 3D**, MCF-7 cells were treated with 0, 4, or 7 μg/ml of bakuchiol for 24 h. For the experiment in **Figure 4C**, MCF-7 cells and MDA-MB-231 cells were treated with ethanol or 7 μg/ml of bakuchiol for 24 h. After the treatment, total RNA of the fish larvae and breast cancer cells was extracted by using the TRIzol reagent in accordance with the manufacturer’s instructions. Total RNA sample was treated with RQ1 RNase-free DNase to decontaminate the genomic DNA, and cDNA was obtained from total RNA by using high capacity RNA-to-cDNA master mix. Real-time PCR was performed with the StepOnePlus^TM^ Real-Time PCR System (Applied Biosystems, Carlsbad, CA, USA), by using the Kapa SYBR FAST qPCR kit, and following the recommendations of the manufacturer. Primers were listed in **Table 1**.

**Table 1:** Primers for quantitative real-time PCR.

	Gene	Forward (5′–3′)	Reverse (5′–3′)
Fish	18S rRNA	CCTGCGGCTTAATTGACCC	GACAAATCGCTCCACCAACT
	ERα	ATTTGGAGGTCCATCCACTG	TGAGTTTGAGCACACGGAAG
	ERβ	AGGAGCATCCAAGGACACAC	GCCGACTTCGTAGCACTTTC
Cell	GAPDH	TCCCTGAGCTGAACGGGAAG	GGAGGAGTGGGTG TCGCTGT
	ERα	AGCTCCTCCTCATCCTCTCC	TCTCCAGCAGCAGGTCATAG
	ERβ	CCTCAAAAGAGTCCCTGGTG	CTTCACACGACCAGACTCCA
	p21	GAGGCCGGGATGAGTTGGGAGGAG	CAGCCGGCGTTTGGAGTGGTAGAA
	p53	CCCCTCCTGGCCCCTGTCATCTTC	GCAGCGCCTCACAACCTCCGTCAT
	ATM	CCCCTTGTGTATGAGCAGGTG	CGGATTATCCTGAGAAGCTC
	ATR	ACATTCCCTGATCCTACATCATG	TTCAATAGATAACGGCAGTCCTG

### Cell Protein Extraction and Western Blot Analysis

**FIGURE 5:**
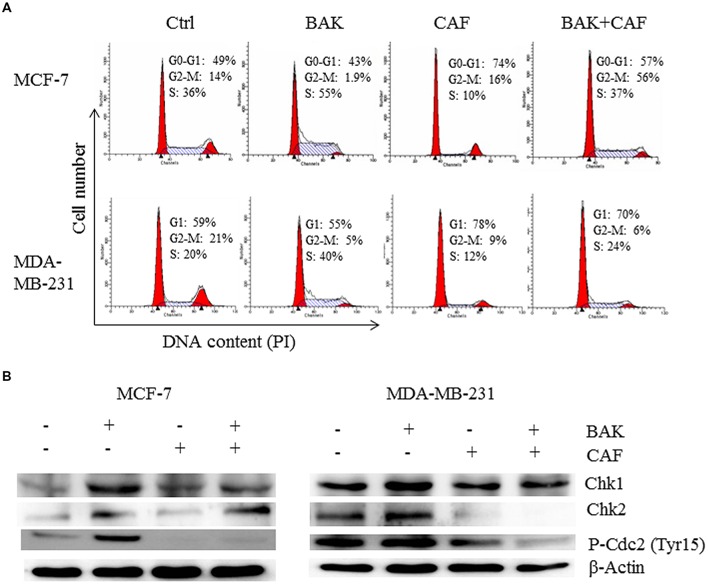
**Caffeine rescued bakuchiol-induced S phase arrest in breast cancer cells.** Cells were treated with ethanol alone or 7 μg/ml of bakuchiol and/or 5 mM of caffeine for 24 h. **(A)** representative cell cycle histograms from three individual experiments of control and treatment with percentage of cell cycle phase indicated. **(B)** Western blot analysis of Chk1, Chk2, P-Cdc2 (Tyr15) in total cell lysates. β-Actin was used as loading control. Ctrl, control; BAK, bakuchiol, CAF, caffeine.

**FIGURE 6:**
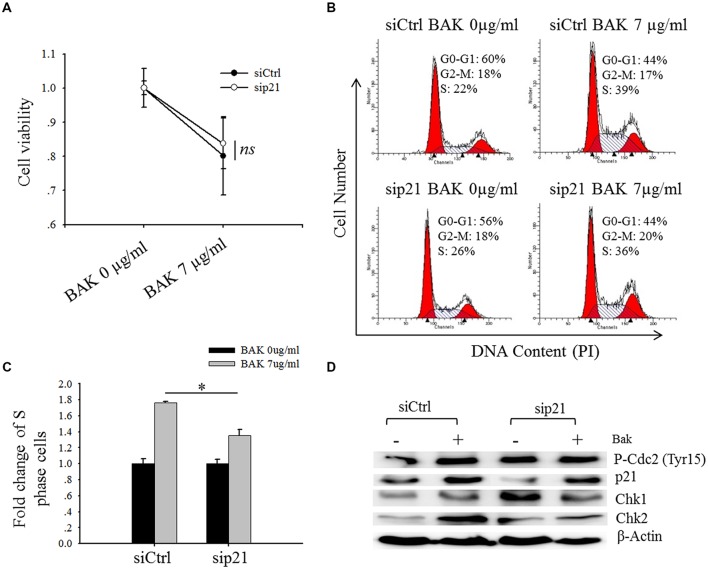
**Knock-down of p21 marginally rescued the bakuchiol-induced S phase arrest.** MCF-7 cells were transfected with siCtrl or sip21, and treated with 0 or 7 μg/ml of bakuchiol for 24 h. **(A)** Cell viability was monitored by MTT assay. The data are expressed as fold change of cell viability with standard deviation (*ns p* > 0.05, student’s *t*-test). **(B)** Cell cycle distribution was studied with PI staining. **(C)** Fold change of cell number in S phase (^∗^*p* < 0.05, student’s *t*-test). **(D)** Western blot analysis of p21, Chk1, Chk2, P-Cdc2 (Tyr15) in total cell lysates. β-Actin was used as loading control. Ctrl, control; BAK, bakuchiol.

**FIGURE 7:**
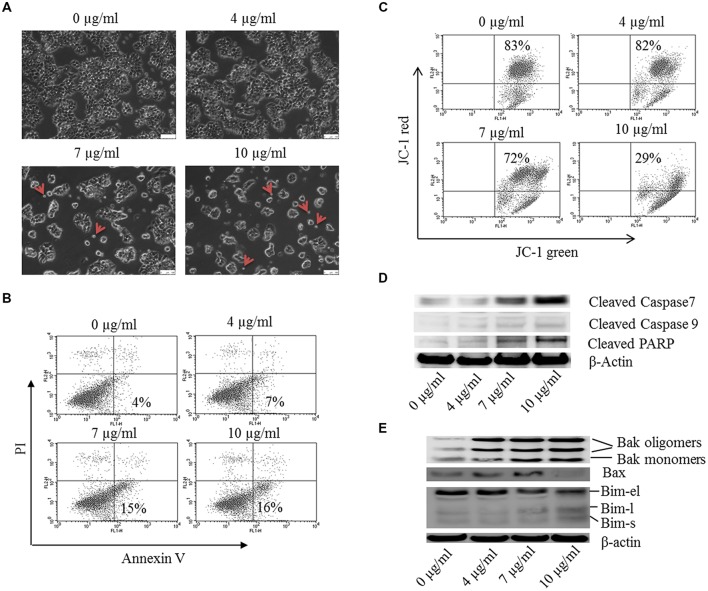
**Bakuchiol induced apoptosis in MCF-7 cells.** MCF-7 cells were treated with 0–10 μg/ml of bakuchiol for 24 h. **(A)** Morphology observation of bakuchiol treated MCF-7 under bright field. Small apoptotic bodies are labeled with red arrows. The white bar indicates 100 μm. **(B)** Representative flow cytometry results of annexin V/PI analysis. **(C)** JC-1 staining for analysis of mitochondrial membrane potential. Western blot analysis of expression levels of **(D)** Caspase family protein, including Cleaved Caspase 7, Cleaved Caspase 9, and Cleaved PARP, and **(E)** Bcl-2 family protein, including Bak, Bax, and Bim. β-Actin was used as loading control.

For the experiment in **Figure 4B**, MCF-7 cells and MDA-MB-231 cells were treated with varying concentrations of bakuchiol for 24 h. For the experiment in **Figure 5B**, MCF-7 cells and MDA-MB-231 cells were treated with ethanol alone or 7 μg/ml of bakuchiol and/or 5 mM of caffeine for 24 h. For the experiment in **Figure 6D**, MCF-7 cells were transfected with siCtrl or sip21, and treated with 0 or 7 μg/ml of bakuchiol for 24 h. For the experiment in **Figures 7D,E**, MCF-7 cells were treated with 0–10 μg/ml of bakuchiol for 24 h. After treatment, the cells were collected and washed twice with ice-cold PBS. Cell pellets were lysed with ice-cold RIPA buffer, which contained 1 mM PMSF and 1% protease inhibitor cocktail. The supernatant was collected and same amounts of proteins were mixed with loading buffer, and heated at 95°C for 10 min before loading onto 10% SDS-polyacrylamide gel. The separated proteins were then transferred to a PVDF membrane (GE Healthcare, Milwaukee, WI, USA), and the membrane was blocked with 5% non-fat milk for 1 h at room temperature. Then, the membrane was probed with an appropriate primary antibody overnight at 4°C. After washing three times with PBT, the membrane was incubated with HRP-conjugated secondary antibody for 1 h at room temperature before visualization with the ECL Prime Western blotting detection system. Antibodies were listed in **Table 2**. Image analysis was performed by using a Fujifilm luminescent image analyzer (Fujifilm Life Science, Stamford, CT, USA).

**Table 2:** List of antibodies.

Antibody	Company	Catalog number
P-Cdc2 (Tyr15)	Cell Signaling Technology	9111
Cyclin B1	Cell Signaling Technology	4138
P-Wee1 (Ser642)	Cell Signaling Technology	4910
Myt1	Cell Signaling Technology	4282
P21 (Waf1/Cip1)	Cell Signaling Technology	2947
Chk1	Cell Signaling Technology	2360
Chk2	Cell Signaling Technology	6334
Cleaved Caspase7	Cell Signaling Technology	9491
Cleaved Caspase9	Cell Signaling Technology	9501
Cleaved PARP	Cell Signaling Technology	5625
Bak	Cell Signaling Technology	6947
Bax	Cell Signaling Technology	5023
Bim	Cell Signaling Technology	2933
β-Actin	Cell Signaling Technology	3700
Anti-rabbit IgG	Cell Signaling Technology	7074
Anti-mouse IgG	Cell Signaling Technology	7076

### Cell Morphology Observation

For the experiment in **Figure 7A**, MCF-7 cells were seeded on sterilized cover slide, and treated with 0–10 μg/ml of bakuchiol for 24 h. After treatment, an inverted microscope (model DMI 3000B, Leica Microsystems, Germany) was used to observe and record the cell morphology.

### Zebrafish Xenografts Establishment and Fish Exposure

Wild-type AB zebrafish embryos were maintained in embryo medium containing PTU at a 0.003% w/v concentration to remove the surface pigmentation. For the zebrafish xenotransplantation, 48 hpf embryos were dechorionated and anesthetized in and 0.004 w/v tricaine before cell injection. Before cell injection, cells were labeled with the Green CMFDA according to manufacturer’s protocol. After staining and washing, cells were loaded into a pulled glass micropipette and attached to an air driven microinjector (Eppendorf FentoJet Express). Approximately 300 cells were injected into the yolk sac of embryos. After injection, embryos were exposed with ethanol (solvent control) or 0.5 μg/ml of bakuchiol. The exposed embryos were maintained for 1 h at 28°C and then placed in to incubator at 34°C for 71 h. Injection was performed using an Olympus SZX12 dissecting microscope, and the xenotransplanted embryos were examined by using a Olympus BX61 flurescence microscope. The integrated fluorescence intensity was quantified by image analysis software ImageJ. All procedures with fish were conducted following the License to Conduct Experiments approved by the Government of the Hong Kong SAR Department of Health [Ref. (15–10) in DH/HA&P/8/2/5 Pt.3].

### Statistical Analysis

Statistical analyses were conducted by using the SPSS 13.0 program. Experiments were carried out three times and the data are presented as mean values ± standard deviation. Student’s *t*-test, one sample *t*-test and one-way ANOVA were used to analyze the data. A *p*-value of <0.05 is considered to be statistically significant.

## Results

### Bakuchiol Exhibits *In Vivo* Estrogenic Activity

Bakuchiol induced GFP expression in the liver of the fish (**Figure 2A**), and LC_50_ and EC_50_ (indicated by GFP induction) of bakuchiol were 1.1 and 0.3 μg/ml respectively (**Figure 2B**). The signal intensity of the induced GFP increased in a concentration-dependent manner (**Figure 2C**). Phytoestrogen usually has weak estrogenic activity. To make the screening results comparable with strong estrogenic compound, the dose-dependent response of transgenic larvae was analyzed with E2 (**Figure 2D**). The GFP signal intensity induced by bakuchiol was compared with the result of the E2 exposure. The GFP intensity of the EC_50_ (0.3 μg/ml) of bakuchiol was comparable to the estrogenicity of 1.6 ppb (∼1.6 μg/l) E2. The results suggested that bakuchiol is a weak estrogen; about 180 times weaker than E2. The expression levels of the ER α and ERβ of fish after 0.5 μg/ml bakuchiol treatment were studied with quantitative real-time PCR. Compared with the control group, both ERα and ERβ exhibit no significant difference in the bakuchiol treatment group the trend of upregulation, although there is (**Figure 2E**), even GFP expression was induced in 94% of the fish after 0.5 μg/ml bakuchiol exposure (**Figure 2B**). Moreover, bakuchiol did not show a significant preference to either ERα or ERβ in whole fish (**Figure 2E**).

### Bakuchiol Induces Cell Growth Inhibition

The cell growth of two breast carcinoma cell lines, MCF-7 and MDA-MB-231, in the presence of various concentrations of bakuchiol, was examined. In the ERα-positive breast carcinoma cell line MCF-7, bakuchiol exerted a biphasic effect on the growth of the cells under the experimental conditions (at 24, 48, and 72 h) – stimulating cellular proliferation at concentrations below 2 μg/ml, but inhibiting cell proliferation in a dose-dependent manner above 2 μg/ml (**Figure 3A**). IC_50_ in MCF-7 cells were 12.1–9.3 μg/ml for 24–72 h exposure. In the ERα-negative breast cancer cell line MDA-MB-231, bakuchiol exhibited a marked growth inhibitory effect in dose- and time-dependent manners (IC_50_: 13.1–8.9 μg/ml). The IC_50_ of resveratrol in breast cancer cells were 2.72–4.15 times of that in bakuchiol (**Figure 3B**), indicating that bakuchiol exhibits stronger anti-breast cancer effect. When MCF-7 cells were co-treated with low dose of bakuchiol (1 μg/ml) and 1 μM ERα antagonist ICI 182780, the proliferative effects of bakuchiol on ERα-positive cells was blocked (**Figure 3C**). The bakuchiol-induced ERα expression was also eliminated by ICI 182780 (**Figure 3C**). Upon treated with high doses (4 and 7 μg/ml) of bakuchiol, the expression levels of ERα were induced and ERβ were suppressed in MCF-7 cells (**Figure 3D**).

### Bakuchiol Induces S Phase Arrest and Expression Level Change of Cell Cycle Regulator

After the cells were treated for 24 h with increasing concentrations (0–10 μg/ml) of bakuchiol, a significant dose-dependent accumulation of cells in S phase in both MCF-7 and MDA-MB-231 (**Figure 4A**) resulted. We then next examined the protein levels of cell cycle regulators (**Figure 4B**). After the cells were treated with 0–10 μg/ml of bakuchiol, the expression level of P-Cdc2 (Tyr15) was up-regulated, accompanied with up-regulation of Myt1 and P-Wee1 (Ser642). In addition, bakuchiol also induced the upregulation of p21 and Cyclin B1. The up-regulation of p21 was also confirmed with real-time PCR (**Figure 4C**). The ATM mRNA expression levels were also significantly induced in both MCF-7 and MDA-MB-231 cells (*p* < 0.05, both, **Figure 4C**). However, there is only an upregulated trend of ATR mRNA level, with no significance. No significant change of p53 mRNA level was observed in both MCF-7 and MDA-MB-231 cells (**Figure 4C**).

### Caffeine Rescues Bakuchiol-Induced S Phase Arrest

When the cells were pre-treated with caffeine, a known ATM/ATR inhibitor, that can inhibits ATM/ATR in MCF-7 and MDA-MB-231 cells, at the concentration of 5 mM (Yu et al., 2013), the bakuchiol-induced accumulation of S phase cells was rescued by caffeine pre-treatment in both MCF-7 and MDA-MB-231 cells (**Figure 5A**). The up-regulated expression levels of P-Cdc2 (Tyr15), Chk1 and Chk2 induced by the bakuchiol treatment were also rescued by pre-treatment with caffeine (**Figure 5B**).

### Depletion of p21 Marginally Rescues S-Phase Arrest Caused by Bakuchiol

When MCF-7 cells were transfected with sip21, the expression level of p21 was down-regulated (**Figure 6D**). Depletion of p21 marginally rescued bakuchiol-induced inhibitory effect, however, the difference of cell viability between bauchiol-treated siCtrl cells and sip21 cells are not significant (*p* > 0.05, **Figure 6A**). In both siCtrl and sip21 transfection cells, bakuchiol induced S phase arrest (**Figure 6B**). The level of S phase cell accumulation was more significant in siCtrl cells than in sip21 cells (*p* < 0.05, **Figure 6C**). Depletion of p21 led to the increased expression of Chk1 and Chk2 (**Figure 6D**). The bakuchiol-induced Chk2 expression was more significant in siCtrl cells than in sip21 cells (*p* < 0.001, **Figure 6D**).

### Bakuchiol Induces Apoptosis of MCF-7 Cells

Morphological change was observed under inverted microscope with bright field (**Figure 7A**). With the bakuchiol concentration increasing, more of the cells became rounded, shrank and were unattached. Besides, more small apoptotic bodies broken from cells can be observed (see red arrow). By using a flow cytometric analysis after annexin V/PI staining, the effects of bakuchiol on breast cancer cell apoptosis were determined. Exposure of MCF-7 cells to bakuchiol elicits a increase in early apoptotic cells, in a dose-dependent manner (**Figure 7B**). Besides, flow cytometric analysis of mitochondrial transmembrane potential Δψm by using JC-1 staining revealed that bakuchiol reduced Δψm of MCF-7 cells dose-dependently (**Figure 7C**). While in MDA-MB-231 cells, bakuchiol did not affect the cell morphology and apoptotic rate under the exposure conditions (data not shown).

The expression levels of Caspase family members and their substrates were determined with Western blot (**Figure 7D**). After 24 h of treatment, the expression levels of cleaved Caspase 7, Caspase 9 and PARP were upregulated by bakuchiol. It has been demonstrated that Bcl-2 family proteins control mitochondrial membrane permeabilization, which is essential for apoptotic regulations. We detected the expression levels of pro-apoptotic Bcl-2 family proteins (**Figure 7E**). The levels of Bim-l, Bim-s, Bak monomers, Bak oligomers and Bax were induced by bakuchiol.

### Bakuchiol Exhibits *In Vivo* Anti-breast Cancer Effect

**FIGURE 8:**
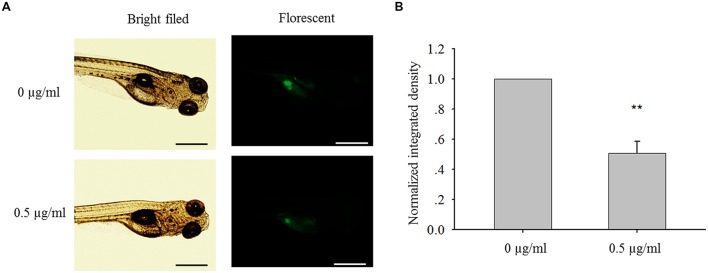
**Bakuchiol inhibited *in vivo* cell mass formation in zebrafish. (A)** ∼300 MCF-7 cells were injected into the yolk sac of 48 hpf zebrafish embryos. 60 embryos were treated with ethanol (solvent control) or 0.5 μg/ml of bakuchiol for 72 h. Triplicates for each condition were performed. Representative bright field and fluorescent images of embryos in both control and treatment groups showing cell mass in the yolk sac area were shown. The black and white bars indicate 500 μm. **(B)** Statistical analysis of integrated fluroscent intensity of cell mass in control and treatment group (^∗^*p* < 0.05, one sample *t*-test). Ctrl, control; BAK, bakuchiol.

After MCF-7 cells were injected into the yolk sac of 48 hpf zebrafish embryos, cell mass formation was observed after 72 h (**Figure 8A**). Compared with control group, bakuchiol significantly inhibited the cell mass formation, which was indicated by the integrated fluorescent signal of CMFDA-labeled cells (*p* < 0.05, **Figure 8B**). Importantly, there was no significant difference in the mortality between control group and treatment group (*p* > 0.05, data not shown).

## Discussion

### Bakuchiol May Act as a SERM and an Anti-breast Cancer Drug

In current study, bakuchiol exhibits estrogenic activity in both *in vivo* and *in vitro* models. Bakuchiol-induced Chg expression in the liver of the transgenic medaka fish suggests that bakuchiol is an ERα agonist in the liver (Chen et al., 2008). When medaka fish were exposed to 0.5 μg/ml of bakuchiol, GFP expression was induced in 94% of the fish. However, the increased expressions of both ERα and ERβ in the whole fish were not significant, and bakuchiol did not show preference to either ERα or ERβ, which all suggest that bakuchiol may act as an ER agonist in some organs and antagonist in other organs in the fish. In previous studies, bakuchiol exhibited different binding abilities to ERα and ERβ in different *in vitro* models, which also implies that bakuchiol has the role of a SERM (Xin et al., 2010; Lim et al., 2011). Another two active ingredients of *Fructus Psoraleae*, named psoralen and isopsoralen, have also been reported with *in vitro* estrogenic activities (Xin et al., 2010; Lim et al., 2011). However, GFP could not be induced by these two compounds in transgenic fish in current study (data not shown). The *in vivo* estrogenic activity of bakuchiol suggests an advantage over other phytoestrogens.

In the ERα-positive breast carcinoma cell line MCF-7, bakuchiol exerts a biphasic effect on the growth of the cells, which is the stimulating of cellular proliferation at low concentrations and inhibiting of growth in a dose-dependent manner at elevated doses. It has been revealed that activation of ERα could promote breast cancer cell proliferation and breast tumor growth (Paruthiyil et al., 2004). Our *in vitro* study confirmed the estrogenic activity of low dose of bakuchiol. Moreover, the biphasic effect of bakuchiol on the growth and ERα expression of MCF-7 cells suggests that bakuchiol is an ERα agonist at low concentrations and antagonist at high concentrations in breast tissue. S phase arrest is observed in both bakuchiol-treated breast cancer cell lines. However, the level of S phase accumulation in ERα-positive MCF-7 cells was higher than that in ERα-negative MDA-MB-231 cells. Besides, bakuchiol only induce apoptosis in MCF-7 cells, not in MDA-MB-231 cells, which all suggests that ERα antagonist effect of bakuchiol contributes to the inhibition of breast cancer cell growth. The bakuchiol-induced ERβ level may also contribute to the inhibitory effects, since binding to ERβ may induce heterodimerization with ERα and hence silence its activation of genes stimulating cell proliferation (Rice and Whitehead, 2006). The anti-breast cancer effect of bakuchiol was further proved in the zebrafish MCF-7 cell xenotransplantation assay. When xenotrasplanted zebrafish embryos were exposed to 0.5 μg/ml bakuchiol, the cell mass was significantly reduced without inducing higher mortality.

The breast cancer cell promoting effect is only achieved at the low concentration of bakuchiol, and the higher concentrations exhibit opposite effect, which is an advantage over estrogen used in HRT. However, high dose of phytoestrogens may have adverse side effects, such as the endocrine disruption of brain and reproductive track (Patisaul and Jefferson, 2010). It is still not clear if higher doses of bakuchiol will exhibit positive effect that overcomes its negative side effect. Thus, bakuchiol may have the potential to be used in HRT and breast cancer treatment, based on its *in vitro* and *in vivo* estrogenic effect and anti-breast cancer effect, but further study is necessary to prove its safety.

### Bakuchiol Induces S Phase Arrest in Breast Cancer Cells Through Inactivation of Cdc2

Cell cycle entry into mitotic phase is initiated by the dephosphorylation of two inhibitory residues, Tyr15 and Thr14 of Cdc2, followed by activation of the Cdc2-Cyclin B1 complex (Zhou and Elledge, 2000). The Wee1 kinase family consists of Wee1 and Myt1, which can phosphorylate Cdc2 to prevent cell entry into mitosis. After treatment with bakuchiol, the expression levels of P-Cdc2 (Tyr15), Myt1 and P-Wee1 (Ser642) were upregulated in breast cancer cells, thus suggesting that bakuchiol-induced Cdc2 (Tyr15) phosphorylation may play a central role in S phase arrest in bakuchiol-treated cells. Based on current data that bakuchiol induced the expression of ATM, Chk1 and Chk2, we hypothesized that bakuchiol-induced S phase arrest is sensed by ATM/ATR. ATM/ATR are members of the phosphoinositide 3-kinase-related kinase family that can be activated to phosphorylate on the serine or threonine residues of their substrates in response to DNA damage or replication blocks (Abraham, 2004). ATM/ATR activates Chk1 and Chk2 (Matsuoka et al., 1998), which are known to phosphorylate Cdc25C at Ser216 in response to DNA damage (Bulavin et al., 2003). Cdc25C is a dual-specificity protein kinase that controls mitotic entry by the dephosphorylation of Cdc2 on both Thr14 and Tyr15 (Coleman and Dunphy, 1994). After pre-treating the cells with 5 mM caffeine, a known inhibitor of ATM/ATR kinases, the S phase arrest and increasing expression levels of Chk1 and Chk2 induced by bakuchiol were abrogated, thus suggesting that ATM/ATR, and Chk1/Chk2 as upstream regulators, control bakuchiol-induced Cdc2 (Tyr15) phosphorylation.

In the bakuchiol-induced S phase arrested cells, the p21 expression level was also upregulated. As discussed above, cell entry mitosis depends on the activation of the Cdc2-Cyclin B1 complex. It has been shown that p21 blocks the activation of the Cdc2-Cyclin B1 complex by preventing the Thr 161 phosphorylation of the Cdc2 subunit (Smits et al., 2000). The induction of p21 depends on the activation of Chk1/Chk2, and this induction could be found in both p53 -dependent and -independent manner (Kastan and Bartek, 2004; Aliouat-Denis et al., 2005). In a negative feedback loop, p21 and p53 can regulate Chk1/Chk2 negatively (Gottifredi et al., 2001; Matsui et al., 2004). Thus, the knock-down of p21 only marginally rescued the bakuchiol-induced S phase arrest.

Both ERα and ERβ can regulate upstream regulators of Cdc2 activation. ERα downregulates the transcription of ATM *via* the activation of miRNAs (Guo et al., 2013). ERα also blocks ATR/CHK1 signal transduction cascade through phosphorylation of TopBP1 protein, thus preventing the enhanced association of ATR with TopBP1 after DNA damage (Pedram et al., 2009). Furthermore, ERα inhibits the expression of p21 by up-regulating miR-17 (Liao et al., 2014). ERβ exhibits opposite effects to ERα. Paruthiyil et al. (2004) reported that ERβ inhibits cell cycle progression by increasing the expression of p21 in MCF-7 cells. The authors also reported that ERβ inactivates Cdc2 by inducing it inhibitors, GADD45A and BTG2 (Paruthiyil et al., 2011). Our results show that high doses of bakuchiol suppressed the ERα mRNA levels and induced ERβ mRNA levels, suggesting that the ER-mediated inactivation of Cdc2 plays an important role in bakuchiol-induce S phase arrest.

### Bakuchiol Induces Apoptosis in MCF-7 Cells via the Intrinsic Mitochondrial Pathway

BH3-only proteins-induced activation of Bax/Bak is essential for mitochondrial-mediated apoptosis. Bim is a BH3-only protein. All the three splice isoforms of Bim (Bim-s, Bim-l, and Bim-el) induce apoptosis via binding to Bcl-2 to the mitochondria to relieve the suppression of Bax/Bak apoptotic effector oligomerization and pore formation at the outer mitochondrial membranes (O’Connor et al., 1998). Once Bax/Bak is activated, cytochrome c is released from mitochondria. In the presence of cytochrome c and dATP, Caspase-9 and Apaf-1 bind to each other via their respective NH2-terminal CED-3 homologous domains, leading to Caspase 9 activation (Li et al., 1997). Caspase 9 propagates the death signal by triggering other caspase activation, such as Caspase 7 (Slee et al., 1999). Activation of Caspase 7 leads to the cleavage and inactivation of PARP (Boucher et al., 2012), thus preventing DNA damage repair.

After treatment with bakuchiol, the expression levels of Bim-l, Bim-s, Bax, and Bak were up-regulated. Besides that, Caspase 7, Caspase 9 and PARP were cleaved. The caspase proteases have been shown to play an important role in the accomplishment of apoptotic morphology. They can target proteins which are involved in the formation and regulation of membrane-associated cortical microfilament cytoskeleton (Saraste and Pulkki, 2000). They can also target proteins which are a part of the cell to cell and cell to matrix attachment (Cardone et al., 1997; Wen et al., 1997). Our results suggested that the induction of apoptosis and apoptotic body formation caused by bakuchiol, is likely through intrinsic mitochondrial pathway.

## Conclusion

In summary, the present study demonstrated that bakuchiol exhibited both *in vitro* and *in vivo* estrogenic activity and anti-breast cancer effect. Bakuchiol exhibited stronger anti-proliferative effects in breast cancer cells than its analog resveratrol. Our data showed that bakuchiol induced S phase arrest in breast cancer cells through inactivation of Cdc2. In parallel, apoptosis analysis showed that bakuchiol induced cell apoptosis via the intrinsic mitochondrial pathway. Our results show the potential of bakuchiol as an anti-breast cancer drug, as well as the potential to be used in HRT for relieving menopausal symptoms, but further study is necessary to prove its efficacy and safety.

## Author Contributions

LL was involved in the project design, carried out most of the experiments, and drafted the manuscript. XC participated in fish maintenance and exposure. CL helped with the zebrafish xenograft establishment. LSL was involved in optimization of the transfection concentration. CM contributed to the experimental design and manuscript preparation. SC contributed substantially to the experimental design, manuscript preparation and submission. All authors read and approved the final manuscript.

## Conflict of Interest Statement

The authors declare that the research was conducted in the absence of any commercial or financial relationships that could be construed as a potential conflict of interest.

## Footnotes

1 http://www.iarc.fr/en/media-centre/pr/2013/pdfs/pr223_E.pdf

## Abbreviations

Chg: choriogenin
DMEM: Dulbecco’s modified Eagle medium
DMSO: dimethyl sulfoxide
dph: days of post-hatching
E2: 17β-estradiol
EC50: effective concentration 50
ER: estrogen receptor
FBS: fetal bovine serum
GFP: green fluorescent protein
hpf: hours of post-fertilization
HRT: hormone replacement therapy
LC50: lethal concentration 50
PBS: phosphate-buffered saline
PI: propidium iodide
PMSF: phenylmethylsulfonyl fluoride
PTU: phenythiourea
RIPA buffer: radio-immunoprecipitation assay buffer
SERM: selective estrogen receptor modulator
